# Supplementary material for: GlomSpheres as a 3D co-culture spheroid model of the kidney glomerulus for rapid drug-screening
Source: Commun Biol. 2021 Dec 2;4:1351. doi: 10.1038/s42003-021-02868-7 (PMC8640035; doi:10.1038/s42003-021-02868-7)
Supplement: Supplementary file 2 — Supplementary Information [file 42003_2021_2868_MOESM2_ESM.pdf]

Supplementary material

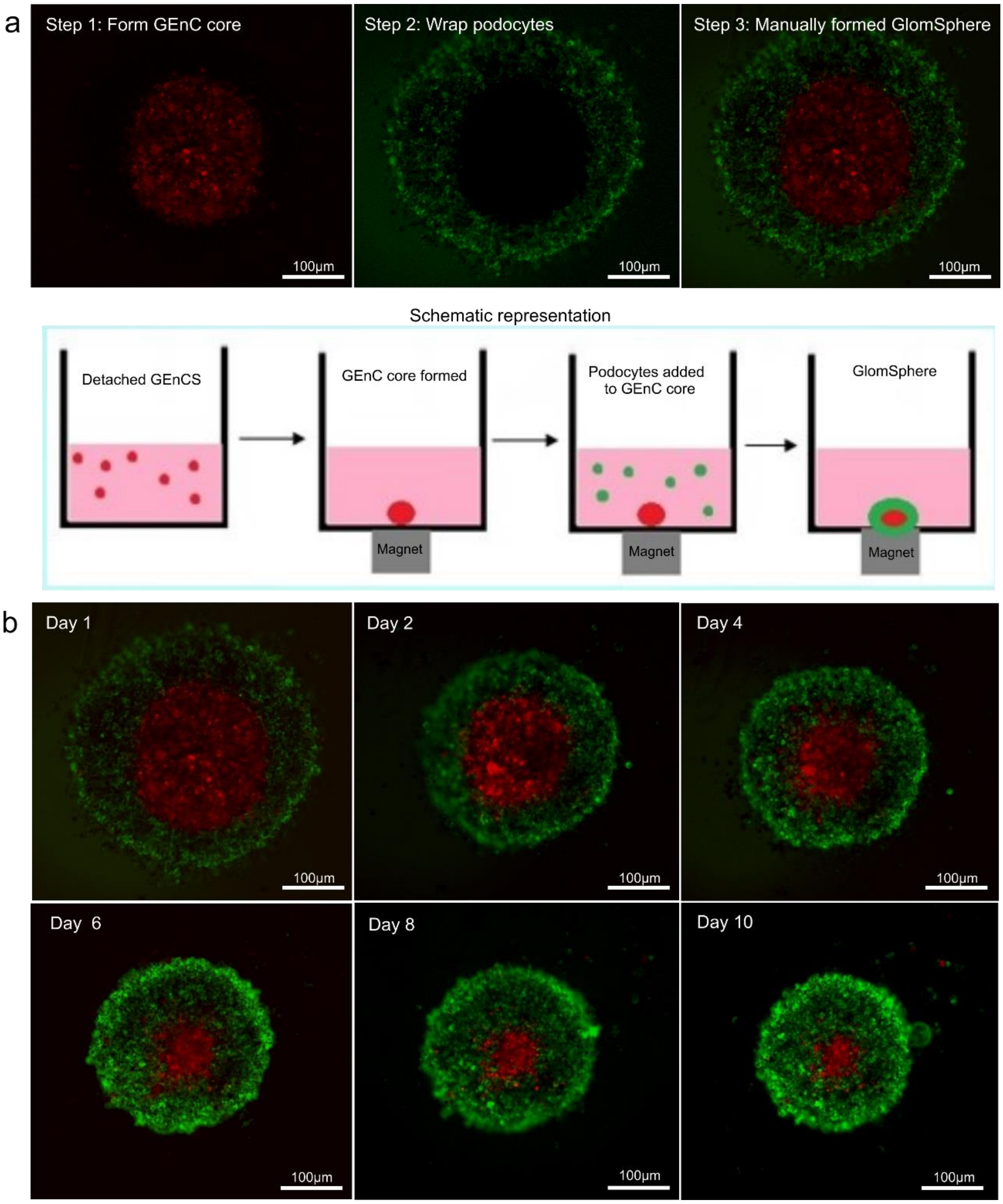

Supplementary Figure 1. Formation and reorganisation of glomerular spheroids. (A) Sequence of spheroid formation. A core of GEnCs (red) is first formed from 5,000 cells. After an hour to stabilise, A peripheral coating of podocytes (green) is then wrapped around the GEnC core, forming a spheroid with a distinct boundary between the two cell types. A schematic representation shows this process more clearly. (B) Differentiation of a glomerular spheroid over 10 days. Peripheral coating of podocytes is shown to migrate around the GEnC core. Overall spheroid diameter is reduced from ~380μm (day 1) to ~220μm (day 10).

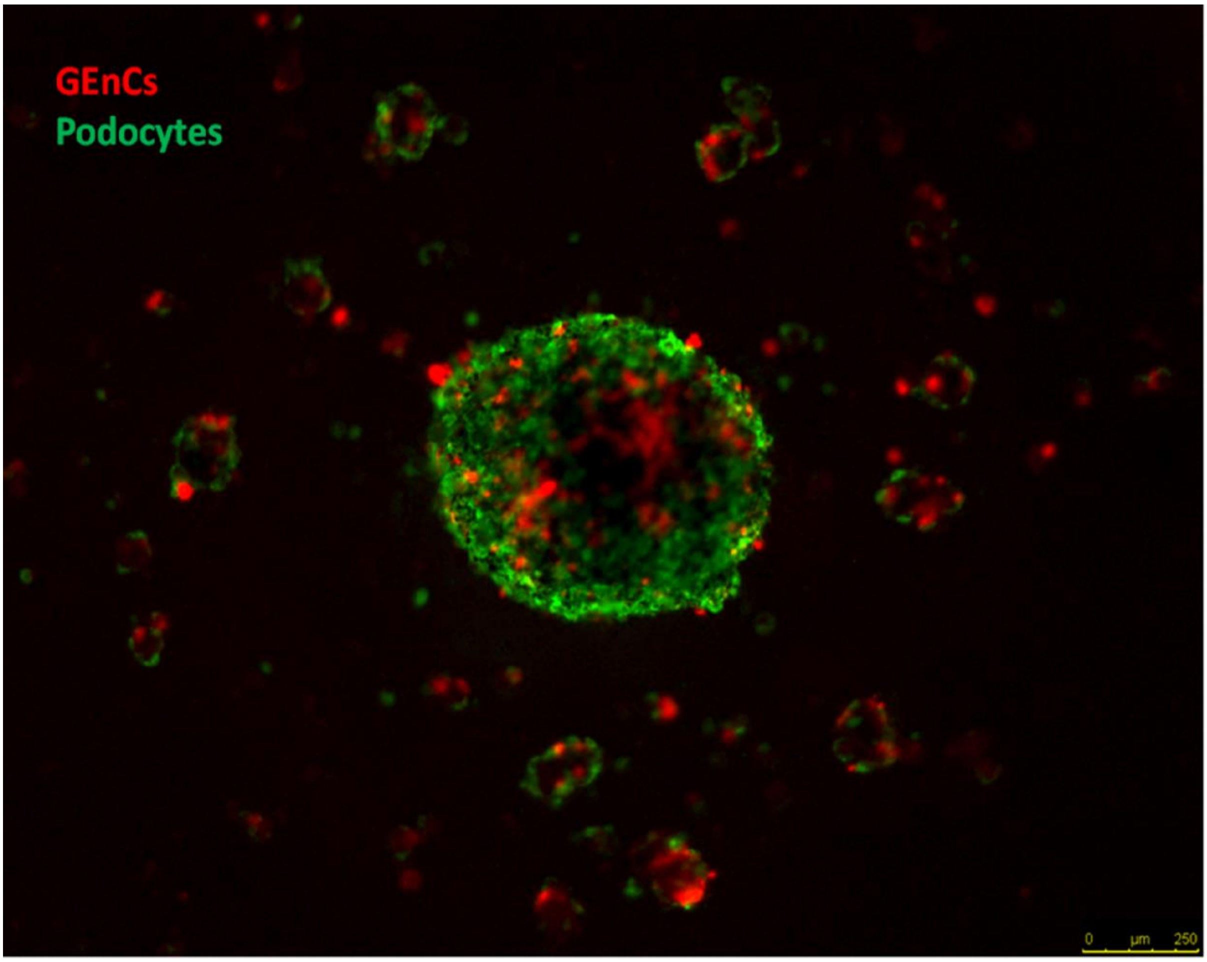

Supplementary Figure 2. **Self-organisation of podocytes (green) and GEnCs (red) after 72 hours of culture.** A large central spheroid is shown to be formed of a core of GEnCs, wrapped by peripheral podocytes. Several smaller spheroids are also shown, which appear to have broken off from the larger structure. Smaller spheroids also appear to have GEnC cores, wrapped by peripheral podocytes. Scale bar = 250µm.

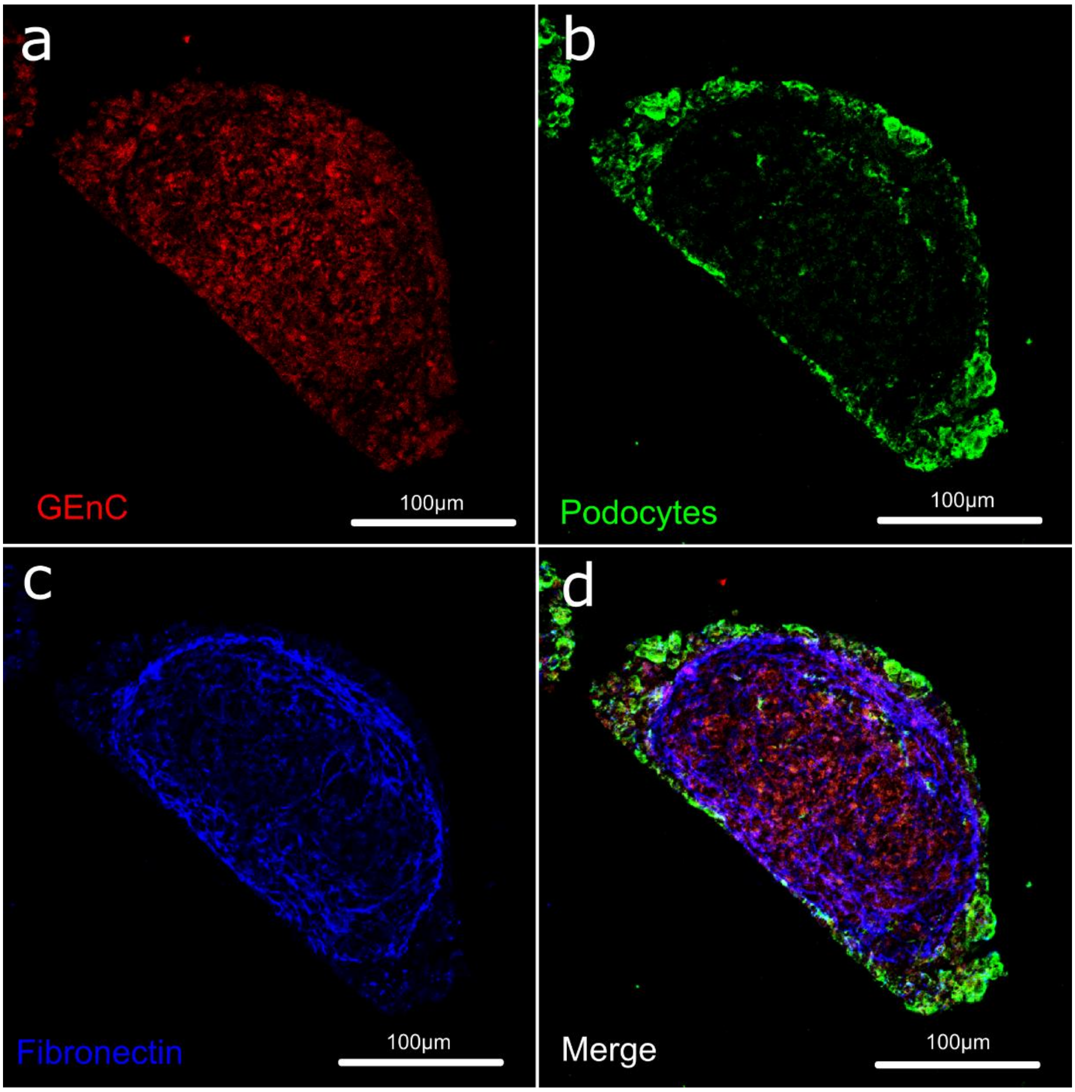

Supplementary Figure 3. Immunofluorescent fibronectin staining of a GlomSphere paraffin section. (a) Central core of GEnCs. (b) Outer layer of podocytes which wraps the endothelial core. (c) Intermediate deposition of fibronectin. (d) Merge, illustrating that whilst fibronectin deposition is concentrated at the podocyte/GEnC interface, it is also spread throughout the spheroid's structure. Scale bars = 100µm.

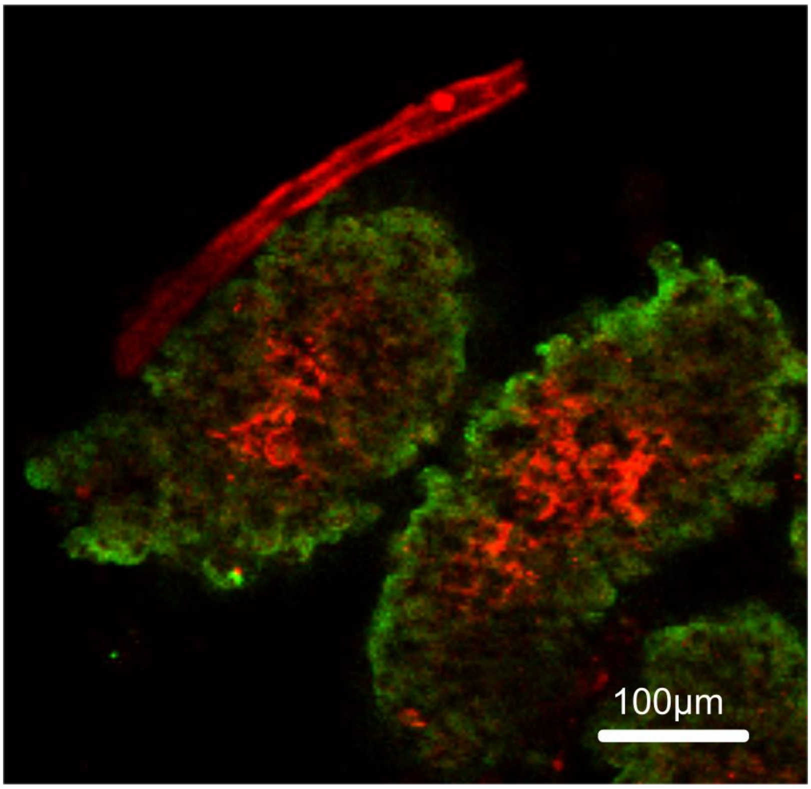

Supplementary Figure 4: GlomSphere stained for nephrin (green) and pecam-1 (red). Nephrin is localised primarily at GlomSphere periphery, whereas pecam-1 is centrally located. The vessel like-protrusion (red) is shown to be pecam-1 positive, indicating that it is endothelial in origin. A hollow lumen can also be seen inside the vessel.

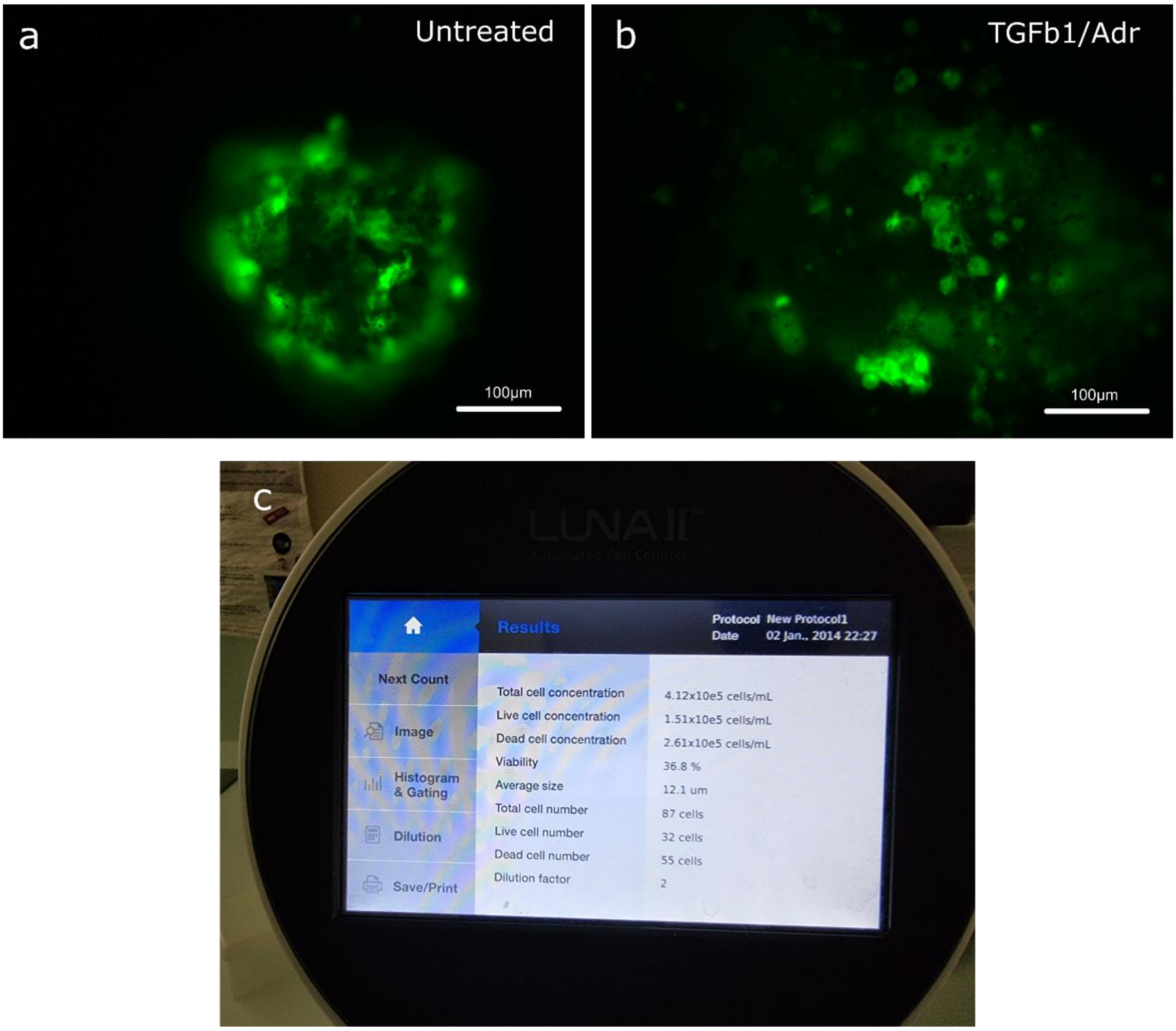

Supplementary Figure 5: Clarification of podocyte loss following injurious stimulation. **a)** The untreated spheroid (left) is shown to have intact podocyte coverage (green), with no cells lost into the surrounding medium. **b)** The TGFβ1 + Adriamycin treated spheroid is shown to have begun losing podocytes into the surrounding medium, which are removed upon changing media. **c)** A cell count of cells lost in this way (with the addition of trypan blue) indicates that these cells were ~36.8% viable at the time of counting.

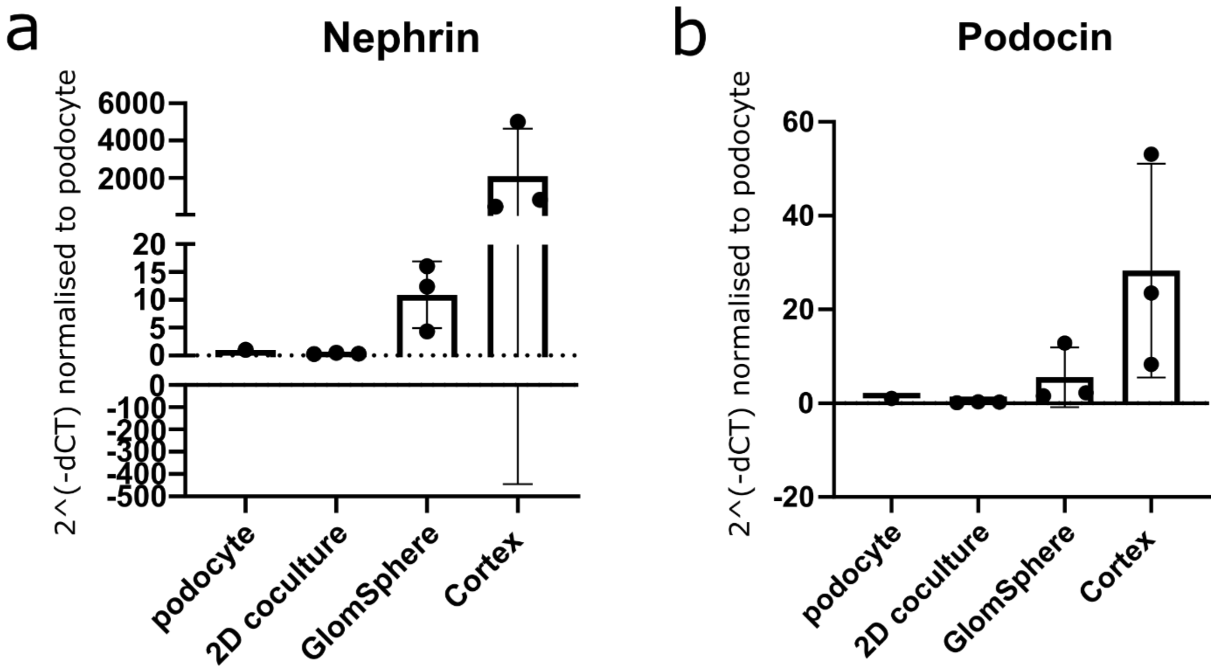

Supplementary Figure 6: Comparison of nephrin and podocin expression in GlomSpheres and human kidney cortex. a) Nephrin expression appears to be much higher in cortex samples than GlomSpheres. Despite this, its expression is negligible in 2D coculture. GlomSphere coculture is therefore restorative of Nephrin expression. **b)** Podocin expression appears to be higher in cortex samples, but GlomSphere expression is within range. This once again shows that GlomSphere expression is restorative of podocin expression.

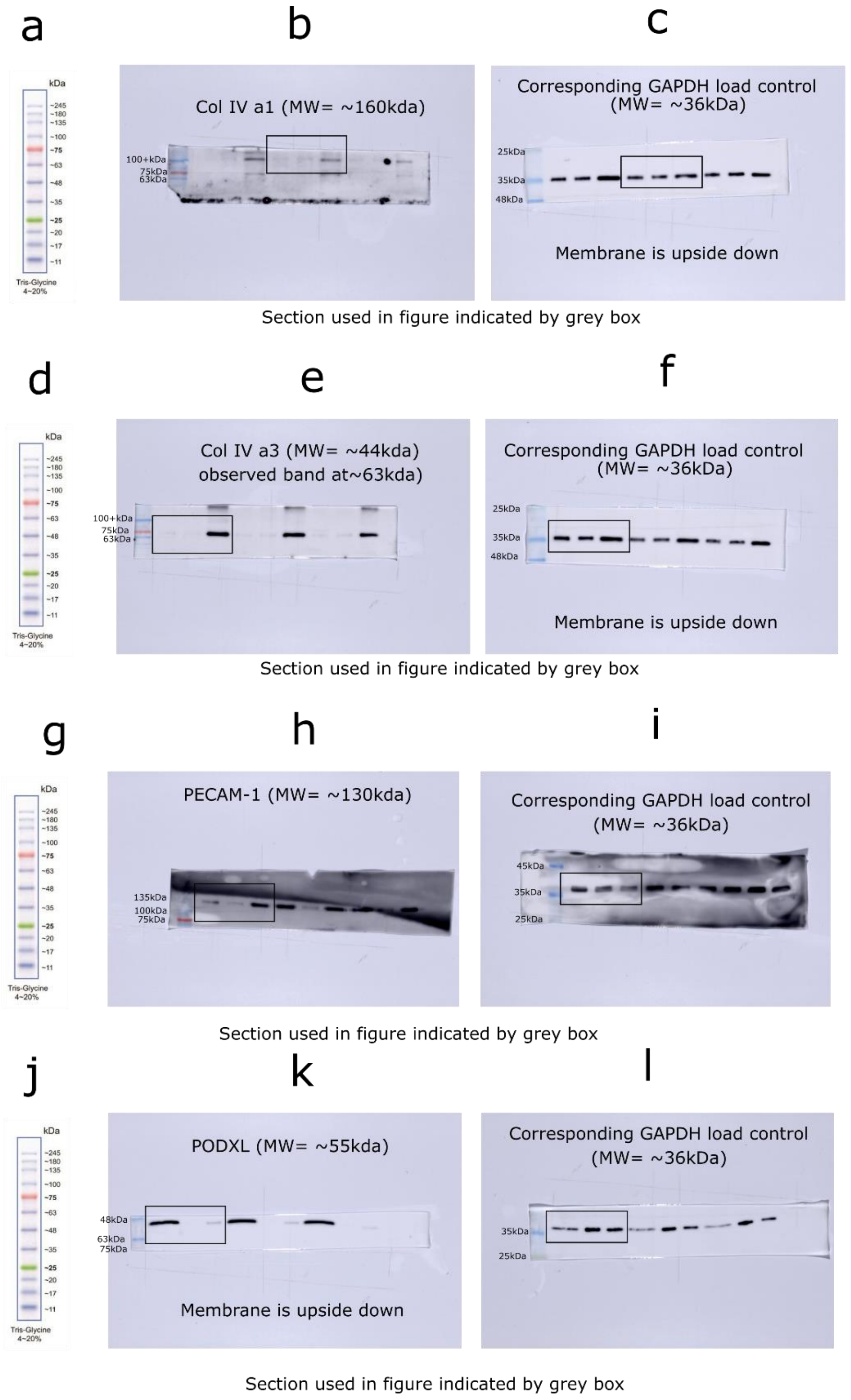

Supplementary Figure 7: Uncropped western blots a) Ladder used for Col IVa1 in fig 2 b) uncropped col IVa1 blot c) corresponding load control for Col IVa1 d) Ladder used for Col IVa3 in fig 2 e) uncropped col IVa3 blot f) corresponding load control for Col IVa3 g) Ladder used for PECAM-1 in fig 4 h) uncropped blot for PECAM-1 i) corresponding load control for PECAM-1 j) Ladder used for PODXL in fig 4) k) uncropped blot for PODXL l) corresponding load control for PODXL
