# Supplementary material for: GlomSpheres as a 3D co-culture spheroid model of the kidney glomerulus for rapid drug-screening
Source: Commun Biol. 2021 Dec 2;4:1351. doi: 10.1038/s42003-021-02868-7 (PMC8640035; doi:10.1038/s42003-021-02868-7)
Supplement: Supplementary file 3 — Description of Additional Supplementary Files [file 42003_2021_2868_MOESM3_ESM.pdf]

Description of Additional Supplementary Files

File name: Supplementary Movie 1

Description: Confocal Z stack slice-by-slice animation. An organized endothelial network of Pecam-1 stained (red) vessel-like structures is shown to be surrounded by Nephrin-expressing podocytes (green).

File name: Supplementary Data 1

Description: Raw data compiled
